# A model of teacher enthusiasm, teacher self-efficacy, grit, and teacher well-being among English as a foreign language teachers

**DOI:** 10.3389/fpsyg.2023.1169824

**Published:** 2023-05-12

**Authors:** Guohua Shao

**Affiliations:** Institute of Physical Education, Inner Mongolia Normal University, Hohhot, Inner Mongolia, China

**Keywords:** teacher enthusiasm, self-efficacy, grit, teacher well-being, EFL, SEM

## Abstract

**Introduction:**

This study aimed to investigate the relationship among teacher enthusiasm and teacher self-efficacy, grit, and teacher psychological well-being among Chinese English as a foreign language (EFL) teachers.

**Methods:**

A sample of 553 Chinese EFL teachers completed self-report measures of teacher enthusiasm, teacher self-efficacy, grit, and teacher psychological well-being. Confirmatory factor analysis was used to confirm the validity of the scales, and structural equation modeling was used to test the hypothesized model.

**Results:**

The results indicated that teacher self-efficacy and grit were positively associated with teacher psychological well-being, providing support for the importance of these teacher characteristics in promoting teacher well-being. Furthermore, teacher enthusiasm was found to have an indirect effect on teacher psychological well-being through the mediation of teacher grit, providing evidence for the importance of teacher motivation and engagement in promoting teacher well-being. The partial mediation model was found to be the best fitting model.

**Discussion:**

These findings have important implications for the development of interventions and programs aimed at promoting teacher well-being in the context of EFL teaching.

## Introduction

As one of the primary decision-makers in language instruction, teachers are believed to play a critical role in the quality of language teaching (Rotgans and Schmidt, 2011). While the knowledge and skills of teachers are important, research has shown that their performance and engagement in the teaching process are influenced by a variety of affective characteristics, personality traits, knowledge, and skills (Dewaele et al., 2018). This implies that teachers’ beliefs, principles, personality traits, and reflection notably impact their instructional performance and engagement in their profession (Kim et al., 2019). According to recent evidence (Chu et al., 2021; Fan et al., 2021; Zhang, 2021), English as a foreign language (EFL) teachers in China might face considerable physical and emotional pressures, particularly in the context of exam-oriented teaching. The ongoing English language education reforms in China (Wen and Zhang, 2020) have led to increased demands on teachers, requiring them to have continuously expanding qualifications (Gao and Xu, 2014; Yuan and Zhang, 2017). In such a complex and stressful environment, it is essential for teachers to possess psychological well-being to overcome the challenges, survive and succeed (Chu et al., 2021; Liu and Chu, 2022).

Teacher well-being as an enabling teacher construct which can mitigate their job stress has been increasingly studied over the past years (Jennings and Greenberg, 2009; Frenzel et al., 2018; Burić, 2019; Li et al., 2022). Having been concerned with the ability to cope with stress, possessing good mental health, having meaning in life, and feeling satisfied with life (Collie, 2022; Nalipay et al., 2022), teacher well-being is associated with student well-being, student learning outcomes, and teachers’ commitment to their profession (Roffey, 2012; Aloe et al., 2014; Chang and Cherng, 2017; Harding et al., 2019). Through well-being, learners and teachers can achieve higher academic achievement and feel positive emotions (Sagone and De Caroli, 2014; Sagone et al., 2018; Greenier et al., 2021; Proietti Ergün and Dewaele, 2021; Xiyun et al., 2022).

Teacher enthusiasm is considered a crucial characteristic of effective and high-quality teaching. It refers to the combination of positive emotional experiences, such as enjoyment in teaching, and the display of these experiences through behaviors (Brophy and Good, 1986; Keller et al., 2016). In addition to influencing students’ academic achievement (Keller et al., 2016; Dewaele and Li, 2021), teacher enthusiasm is positively correlated with students’ motivation, affective, and behavioral outcomes (Patrick et al., 2000; Frenzel et al., 2009; Burić, 2019). This construct has also been identified as one of the characteristics that distinguish good teachers as well as a key indicator of instruction’s quality which might contribute to teachers’ health, happiness, and well-being (Keller et al., 2016; Lazarides et al., 2018).

The concept of self-efficacy refers to one’s perception in his or her ability to plan and execute the actions necessary to produce a given outcome, and the concept of teacher self-efficacy is characterized as a teacher’s belief in the ability to affect student learning outcomes (Bandura, 1997). Teachers may hold many beliefs, but few are more significant to their behavior and activities in class than their sense of efficacy (Zee et al., 2018a). There have been several theoretical theories suggesting that teachers’ self-referential capability judgments, or teacher self-efficacy, play a key role in determining what activities to engage in the classroom, how much effort is expended in those activities, and how well teachers persist in challenging classroom environments (Tschannen-Moran and Hoy, 2001; Zee et al., 2018b).

Moreover, studies have indicated that educators who report higher levels of self-efficacy tend to implement a wider variety of teaching techniques, frequently customize their approach, and display greater attentiveness to students’ cues, requirements, and anticipations than those who lack such convictions (Thoonen et al., 2011; Martin et al., 2012; Zee et al., 2018a). In light of these findings, it may not be surprising that teachers’ well-being may also be influenced by their efficacy perceptions (Helms-Lorenz and Maulana, 2016; Huang et al., 2019). Although English language teachers’ careers may be successful in a number of ways, depending on several factors such as education, personality, connections, timing, willingness to take risks, and luck (Derakhshan et al., 2022), the role of grit is an important factor that should not be undervalued (Sudina et al., 2021a). Grit is a non-cognitive variable characterized by perseverance and a passion for long-term goals. Teachers who have greater passions for their students and their teaching activities are likely to be happier and possess greater well-being (Bashant, 2014; Maiers and Sandvold, 2017; Sudina et al., 2021a,b; Azari Noughabi et al., 2022).

Although some studies have examined the associations among teacher enthusiasm, teacher self-efficacy, grit, and teacher well-being with one another or with other teacher intrapsychic constructs (e.g., Helms-Lorenz and Maulana, 2016; Fabelico and Afalla, 2020; Dewaele and Li, 2021; Yang, 2021; Azari Noughabi et al., 2022; Billett et al., 2022; Khammat, 2022; Liang et al., 2022; Wang et al., 2022), no single study has ever explored the interconnections among these constructs in the context of Chinese EFL teachers. Therefore, the purpose of this study is to investigate the relationship among teacher enthusiasm, teacher self-efficacy, grit, and teacher well-being among Chinese EFL teachers.

With the increasing demands and pressures on teachers in the Chinese EFL context, especially due to English instruction reforms, understanding the factors that contribute to teacher well-being and performance has become increasingly important (Liu and Chu, 2022). In recent years, the Chinese government has implemented several English instruction reforms aimed at improving the quality of English education in the country. These reforms include changes to the English language curriculum, teaching materials, and teaching methods, leading to introducing new national standards for English language teaching, emphasizing the development of communicative competence, critical thinking, and problem-solving skills among learners (Gao et al., 2014). Additionally, there has been a shift toward more student-centered teaching approaches and the integration of technology in the classroom. The reforms also emphasized the importance of teachers’ professional development and encouraged them to participate in training programs to improve their teaching skills (Wen and Zhang, 2020). However, the implementation of the reforms has been challenging, as teachers face a range of difficulties, including large class sizes, limited resources, and pressure to meet high expectations from students, parents, and administrators. As such, investigating factors that can promote teachers’ well-being is critical to improving the quality of English instruction in China. Moreover, the four variables selected in this study are theoretically and conceptually connected, as they all relate to teachers’ emotional experiences and perceptions of their work. By exploring the interplay among these constructs, this study intends to provide a more illustrative understanding of the factors contributing to teacher well-being in the EFL context, which can inform the development of targeted interventions and support systems for these teachers.

## Literature review

### Well-being

Research has shown that teachers’ emotions are a key factor in shaping their students’ academic outcomes (Day, 2004; Burić, 2019). While positive emotions can enhance students’ learning experiences, negative emotions can have the opposite effect (Yin and Lee, 2012; Taxer and Frenzel, 2015; Burić, 2019). Moreover, recent research has shifted toward exploring positive health-related factors such as well-being, satisfaction, and resilience, rather than solely focusing on negative factors such as anxiety and burnout. This shift is due to the emergence of positive psychology in the late 20th century, which highlights the importance of promoting well-being as a way to improve overall functioning and life satisfaction (Gallagher and Lopez, 2009).

As a positive psychology construct, teacher well-being is a complex and multifaceted construct which encompasses professional fulfillment and happiness, including general satisfaction, domain-specific well-being, and affective experiences (Diener, 2000; Diener et al., 2017). Research in this area often explores the factors that influence job satisfaction and overall well-being, including personal characteristics and school-related variables (Collie and Martin, 2017; Li et al., 2022). Furthermore, well-being can be conceptualized in different ways, with some scholars emphasizing the importance of acceptance of oneself, purpose in life, personal growth, positive relationships with others, autonomy, and knowledge of the environment (Ryff, 1989). Others argue that well-being is best understood through the PERMA model, which highlights the importance of positive emotion, engagement, relationships, meaning, and accomplishment in making life fulfilling and meaningful (Seligman, 2012; Mercer and Gregersen, 2020).

Teachers’ well-being has been found to have a significant impact on their interactions with students, with those who experience well-being being more engaged and caring, while those suffering from emotional exhaustion are more critical (Mercer and Gregersen, 2020). In the literature, two approaches have been used to analyze well-being: subjective well-being and psychological well-being, which are measured using hedonic and eudaemonic measures, respectively. While these approaches are useful for understanding the different aspects of well-being, in reality, well-being is likely a combination of both (Mercer, 2020).

Recent research has emphasized the importance of teacher well-being as a positive health-related factor that can benefit EFL teachers in various ways (Kern et al., 2014; Talbot and Mercer, 2018; Turner and Theilking, 2019). Psychological well-being, which encompasses physical and mental health, life satisfaction, and work fulfillment, is a more sustainable sense of meaning than self-actualization (Gregersen et al., 2020; Mercer, 2021). For EFL teachers, well-being involves maintaining a positive and content state of mind despite professional challenges, fostering social connections, trust, and wellness, and experiencing a sense of fulfillment, satisfaction, purpose, and happiness in collaboration with colleagues and students (Acton and Glasgow, 2015; Mercer, 2020; Solarte, 2021). Although well-being is not fixed and can be influenced by various factors, such as gender, experience, children, or interpersonal behaviors (Van Petegem et al., 2005), teachers can tangibly demonstrate their well-being through positive interactions with students, such as smiling, using humor, providing feedback, and designing engaging activities (Dewaele and MacIntyre, 2014; Turner and Theilking, 2019; Solarte, 2021).

Understanding the antecedents of teacher well-being has become increasingly important in mainstream education due to its impact on teacher retention, student behavior, and academic performance (MacInerney et al., 2018; Corcoran and O'Flaherty, 2022). For instance, Li et al. (2022) recommended the development of resources to help teachers who experience positive emotions in school cope with work challenges and maintain positive evaluations of their teaching work. Conversely, teachers who experience negative emotions in school may suffer from emotional exhaustion or disengagement in their classroom, leading to a cycle of negative emotions. Therefore, investigating the factors that contribute to teachers’ well-being is critical for both teachers and students, and can have positive implications for the education system as a whole.

Several studies have highlighted the crucial role that well-being plays in shaping teachers’ psychological state and pedagogical practices (Seligman, 2018; Manasia et al., 2020). To better understand the factors that contribute to well-being, Oxford’s (2016) EMPATHICS model of positive psychology proposes nine key elements. However, EFL teachers face several challenges that can negatively impact their well-being, including extreme work conditions, compulsory educational reforms, limited autonomy, and various learning contexts (Gregersen et al., 2020; Jin et al., 2021). In response, some researchers have focused on developing strategies to enhance EFL teachers’ emotional intelligence, instructional competencies, and professional well-being (Dewaele et al., 2018). Other studies have shown that EFL teachers’ well-being is positively associated with the joy of teaching jubilant learners (Greenier et al., 2021) and pleasant emotions, engagement, positive connections, and accomplishment (MacIntyre et al., 2019). However, severe workloads and limited financial assistance continue to pose major threats to EFL teachers’ well-being (Talbot and Mercer, 2018). In fact, Talbot and Mercer's (2018) study revealed that intense workload was a common issue among EFL teachers, and temporary work status and limited language ability further compounded their challenges.

Taken together, teacher well-being in the context of EFL is of much significance because teacher burnout and stress are common in this field due to a variety of factors, such as heavy workloads, low job security, and cultural and linguistic barriers (Talbot and Mercer, 2018; Wang et al., 2022). In the EFL context, teachers who have high levels of well-being are more likely to stay in the profession and provide high-quality instruction to their students (Sudina et al., 2021a). Therefore, it is important to examine the factors that promote teacher well-being in order to develop effective interventions to support EFL teachers.

### Teacher enthusiasm

Teacher enthusiasm has been conceptualized in various ways in the literature. Initially, it was viewed as a form of instructional behavior, encompassing nonverbal cues such as smiling, eye contact, gesturing, and facial expressions (Song, 2022). However, this led to a one-dimensional perception of enthusiasm, where it was only considered as teacher enjoyment (Kunter et al., 2011; Song, 2022). A more current understanding of teacher enthusiasm posits that it is a quality of effective teaching, which impacts students’ scholarly achievement by demonstrating increased energy and interest in the subject matter and its presentation in dynamic and engaging ways (Song, 2022). In this view, enthusiasm refers to teachers’ ability to express themselves and communicate to their students the importance and value of the material being taught (Wang and Guan, 2020; Song, 2022). On the other hand, a bi-dimensional perspective regards teacher enthusiasm as an emotional-behavioral trait requiring personal willingness and emotion (Kunter et al., 2011). Specifically, in the context of classroom teaching, teacher enthusiasm is defined as an affective experience of enjoyment, excitement, and pleasure, accompanied by expressive behaviors (Kunter et al., 2011; Song, 2022).

Importantly, the learning context plays a crucial role in shaping the meaning and significance of teacher enthusiasm. In foreign language teaching, for example, teacher enthusiasm holds even greater significance as it can prove to be more rewarding and valuable (Dewaele and Li, 2021). Given the complexity and difficulty of teaching and learning foreign languages, enthusiasm and positive attitudes, interest, and passion for learning are essential to keep language learners on track during the tedious and exhausting process of language learning (Dörnyei and Ushioda, 2011). Moreover, as Kunter et al. (2008) noted, enthusiasm might be considered an intrinsic aspect of instructors’ motivation that is reflected in high-quality instructional behavior, but its importance in different learning contexts may vary. Therefore, it is important to consider the learning context when examining the impact of teacher enthusiasm on students’ academic performance, attitudes, interests, motivations, and learning outcomes (Keller et al., 2013; Lazarides et al., 2018; Burić, 2019).

Several scholars have attempted to define teacher enthusiasm, with varying perspectives on its nature. Orosz et al. (2015) acknowledged the difficulty in accurately defining enthusiasm, and proposed two possible definitions: behavior (such as inspiring gestures, facial expressions, and vocal delivery) or an internal state that is a personal trait of the individual. Building on this, Keller et al. (2013) identified eight characteristics of enthusiastic teachers, including the use of demonstrative gestures, facial expressions, and instructive language, as well as a willingness to welcome students’ thoughts and emotions while maintaining overall energy throughout the class. Similarly, Kunter et al. (2008) suggested that teacher enthusiasm can be inferred from evaluations of instructional activities, such as reflecting on the quality of learning materials or expressing interest in the subject. Notably, Mitchell (2013) distinguished between subject matter enthusiasm and subject matter enthusiasm for teaching, with examples of the former including prep work before instruction, verbalizing personal experiences, and planning meaningful educational experiences. Wenström et al. (2018) also found that teacher enthusiasm was reflected in a desire to improve knowledge and abilities in the profession. Therefore, instructors’ enthusiasm for the subject matter can be demonstrated through embracing students, verbal and nonverbal interactions, acknowledging students’ thoughts and feelings, and providing positive feedback on their activities during class.

Overall, teacher enthusiasm is an essential component of effective teaching in the EFL context since it helps to motivate students and create a positive learning environment (Dewaele and Li, 2021; Peng, 2021). Enthusiastic teachers are more likely to engage students in the learning process and facilitate their language acquisition (Papi and Abdollahzadeh, 2012). Furthermore, teacher enthusiasm has been linked to positive outcomes such as job satisfaction, commitment, and performance (Dewaele and Li, 2021). Hence, exploring the factors that contribute to teacher enthusiasm in the EFL context is important for improving the quality of instruction and enhancing the overall learning experience of students.

### Teacher self-efficacy

Self-efficacy is a critical construct in the field of education, particularly among teachers. Bandura (1997) defines self-efficacy as an individual’s belief in their capacity to plan and carry out actions necessary to accomplish particular objectives. Specifically, in the context of teaching, self-efficacy pertains to teachers’ confidence in their ability to implement instructional strategies, increase student engagement, and develop classroom management skills (Kazemkhah Hasankiadeh and Azari Noughabi, 2022). Research has shown that self-efficacy has a significant impact on human function as it influences behavior directly and indirectly, impacting factors such as objectives, outcome expectations, and perception of opportunities and obstacles in the social environment (Bandura, 2006). Additionally, people can nurture their self-efficacy, and their perceptions of efficacy have an impact on cognitive patterns, behaviors, and emotional arousal. Luszczynska et al. (2005) distinguish between general self-efficacy, which refers to the belief in one’s ability to handle a range of challenging or difficult tasks, and specific self-efficacy, which is focused on the specific task being performed. Bandura (1986) notes that self-efficacy is linked to global self-images and is based on a positive self-image supported by self-worth. Also, motivation is also considered an important factor for achieving academic success, and researchers have found that motivation and self-efficacy are closely intertwined (Hsieh and Schallert, 2008). It is worth noting that the construct of self-efficacy and motivation are two distinct but interrelated constructs in educational research. Although self-efficacy is defined as an individual’s belief in their ability to perform a specific task, motivation is the driving force that compels an individual to take action toward a goal (Hsieh and Schallert, 2008). However, both constructs are closely related as individuals with high self-efficacy are more likely to be motivated to engage in a task and persevere in the face of challenges (Zimmerman, 2000).

Several studies have explored the relationship between teacher self-efficacy and other constructs related to teacher well-being, such as exhaustion, work satisfaction, and burnout. Federici and Skaalvik (2012) found that self-efficacy had a negative relationship with exhaustion, but a positive association with motivation and work satisfaction. Similarly, Malinen and Savolainen (2016) reported an inverse link between teachers’ self-efficacy and exhaustion. In a recent study, Fathi et al. (2021) investigated the causal relationships among EFL teachers’ self-efficacy, reflection, and burnout in the EFL context. They found that emotion regulation could moderate the effects of reflection and self-efficacy on burnout among EFL teachers. Notably, self-efficacy is a crucial predictor of academic performance, and studies have shown that it has a stronger impact on performance than other factors such as motivation alone (Pajares and Kranzler, 1995; Dobbins, 2016). By integrating the concept of self-efficacy into a psychological approach, individuals can enhance their belief in their ability to achieve their goals, which can positively influence their behaviors, emotions, and cognitive patterns (Bandura, 2006).

Research has shown that teacher efficacy influences student efficacy and achievement, as well as teacher perseverance, enthusiasm, resilience, commitment, and sense of job satisfaction (Bandura, 1997; Tschannen-Moran and Hoy, 2001; Tschannen-Moran and Barr, 2004; Dobbins, 2016). In fact, effective instructors accept responsibility for poor student performance and modify their actions to improve outcomes (Bandura, 1997). However, low self-efficacy among teachers can create a negative cycle of poor student achievement and decreased teacher efficacy (Tschannen-Moran et al., 1998; Dobbins, 2016). This can lead to greater work stress and lower job satisfaction among teachers (Betoret, 2006). Recent studies have investigated the relationship between self-efficacy, classroom management, and job satisfaction among teachers. For example, Sulla and Rollo (2023) found that a short course on classroom management led to increased rates of praise from Italian primary school teachers and improved on-task behavior of their students. This suggests that self-efficacy in classroom management can have a positive impact on both teacher job satisfaction and student behavior. These findings support our hypothesis that teacher self-efficacy is an important factor in promoting teacher well-being, particularly in the EFL context. By enhancing their self-efficacy in areas such as classroom management, teachers may feel more confident and satisfied in their roles, leading to improved overall well-being.

As far as EFL context is concerned, teacher self-efficacy is a significant construct in the EFL context because it has been shown to be a strong predictor of teacher well-being, engagement, and burnout (Tschannen-Moran and Hoy, 2007; Skaalvik and Skaalvik, 2017; Bing et al., 2022; Xiao et al., 2022; Xiyun et al., 2022). Self-efficacious teachers are more likely to set challenging goals, persist in the face of obstacles, and use effective teaching strategies (Bandura, 1997). As such, it is important to identify the antecedents and correlates of teacher self-efficacy in the EFL context.

### Teacher grit

Teacher grit is an important construct that has garnered increasing attention in the educational literature due to its positive association with academic outcomes (Fernández Martín et al., 2020; Santana-Monagas and Núñez, 2022). Defined as a combination of perseverance and passion toward achieving long-term goals (Kwon, 2021; Santana-Monagas and Núñez, 2022; Teimouri et al., 2022), grit can help individuals overcome obstacles and setbacks without giving up or burning out (Duckworth et al., 2007).

Teacher grit involves the same characteristics of persistence and passion toward achieving long-term goals, but from the perspective of the educator (McCain, 2017). The concept of grit is multidimensional, with two main facets: consistency of interest and perseverance of effort (Wang et al., 2021a,b). Consistency of interest involves maintaining one’s interest in achieving goals despite obstacles, while perseverance of effort refers to the inclination to work hard and exert effort when faced with difficulties (Li, 2020). Despite the validation of grit with a two-factor structure, there are several studies that have reported a one-dimensional structure (González et al., 2020; Postigo et al., 2021), which raises questions about the clarity of grit as a construct. This controversy has recently been recognized by Duckworth et al. (2021), who suggested that the interpretation of the grit construct may depend on the context in which it is being measured. Therefore, the findings related to grit should be interpreted with caution, and further research is necessary to establish the most appropriate method of measuring grit in different contexts.

Recent studies have highlighted the importance of teacher grit in language classrooms. Kazemkhah Hasankiadeh and Azari Noughabi (2022) found that teachers’ perspectives on their own grit were associated with their determination to succeed despite setbacks. Additionally, Zeng et al. (2019) suggested that teachers who exhibited higher levels of grit were more likely to engage in effective teaching behaviors and promote students’ academic achievement. Furthermore, grit is a malleable construct that can be improved through training and intervention in the school setting (Clark and Malecki, 2019). As such, teachers can play an instrumental role in helping students develop grit and overcome potential challenges during the language learning process (Sudina and Plonsky, 2021).

Grit is distinct from other constructs such as self-efficacy and self-concept, as it is associated with a more goal-oriented approach (Santana-Monagas and Núñez, 2022). While self-efficacy and self-regulation can promote instructors’ self-determination and persistence in dealing with daily failures, grit is characterized by a willingness to exert effort and work hard when faced with obstacles and setbacks (Skaalvik and Skaalvik, 2017; Kazemkhah Hasankiadeh and Azari Noughabi, 2022). As Sudina et al. (2021a) noted, teacher grit is linked to maintaining passion for the profession and exhibiting greater resilience in the face of setbacks. In a study by Skaalvik and Skaalvik (2014), it was found that instructors with greater self-efficacy are more likely to be committed and motivated in their work. Moreover, research by Duckworth et al. (2009) found that grit and life satisfaction were important indicators of successful teaching, and gritty teachers are more likely to enjoy their work and perform well in the classroom, as noted by Maiers and Sandvold (2017). Additionally, individuals with grit tend to exert themselves in the pursuit of goals that inspire them and stay focused on them over time, despite setbacks, challenges, or fatigue (Verner-Filion et al., 2020).

Gritty teachers, as described by Duckworth et al. (2009), experience greater job satisfaction and fulfillment. Furthermore, gritty teachers exhibit greater enthusiasm, commitment, and engagement in the classroom (Maiers and Sandvold, 2017), which may lead to better student outcomes. In the EFL context, however, teachers may face various challenges such as job instability, low income, cultural and linguistic barriers, increasing workload, and a lack of confidence, all of which can test their persistence and compassion (Sudina et al., 2021a). Despite these challenges, teachers who can maintain their enthusiasm for their work and navigate obstacles without giving up demonstrate the persistence that Duckworth et al. (2007) describe as grit. Moreover, the positive association found between teachers’ grit and their growth mindset, as noted by Derakhshan et al. (2022) and Zeng et al. (2019), can enhance their mental well-being and help them cope with pressure. Therefore, cultivating grit in teachers can be beneficial for both teachers and students.

Taken together, teacher grit is an important construct in the EFL context because language learning can be a challenging and lengthy process (Sudina et al., 2021a,b; Liu et al., 2023). EFL teachers who possess grit are better equipped to overcome obstacles and persist in their efforts to help their students succeed (Sudina and Plonsky, 2021). Moreover, research has shown that teachers who have high levels of grit are more likely to enjoy their work and exhibit greater enthusiasm and commitment (Duckworth et al., 2009; Maiers and Sandvold, 2017). Therefore, examining the constructs that contribute to teacher grit in the EFL context, such as training and intervention programs, can help to develop effective strategies to support EFL teachers in their pursuit of professional and personal growth.

### The hypothesized model

Given the empirical and theoretical background reviewed above, it is hypothesized that several interrelated factors might contribute to teacher well-being in the EFL context. More precisely, a structural model was suggested in which it was hypothesized that teacher enthusiasm and teacher self-efficacy are both directly related to teacher well-being, and that teacher self-efficacy also has indirect effects on teacher well-being through its influence on teacher enthusiasm and teacher grit. The following hypotheses guide this research:

*H1*: Teacher enthusiasm positively influences teacher well-being.

There is a bulk of research suggesting that teacher enthusiasm can play a significant role in promoting teacher well-being (Day and Qing, 2009; Bardach et al., 2022; Billett et al., 2022). Enthusiastic teachers are often described as having high levels of energy, excitement, and engagement in their work (Kunter et al., 2011; Burić and Macuka, 2018). This enthusiasm can have a positive impact on their emotional state, helping to promote positive emotions such as joy, interest, and contentment (Sutton, 2007; Keller et al., 2014). Furthermore, enthusiasm is also associated with a sense of meaning and purpose in one’s work (Federici and Skaalvik, 2012; Lazarides et al., 2018). Teachers who are enthusiastic about their profession may see their work as a calling rather than just a job, which can contribute to greater job satisfaction and well-being (Goddard et al., 2004; Burić and Moe, 2020). Enthusiastic teachers may also experience a greater sense of fulfillment and personal growth from their work, which can enhance their overall well-being (Bieg et al., 2022). In light of these arguments, it is theoretically justifiable to hypothesize that teacher enthusiasm has a positive influence on teacher well-being.

*H2*: Teacher self-efficacy has a positive effect on teacher well-being.

A substantial amount of research has reported that teacher self-efficacy, or the belief that one has the ability to successfully perform teaching-related tasks and responsibilities, is associated with positive outcomes in the teaching profession (Helms-Lorenz and Maulana, 2016; Billett et al., 2022). In particular, there is strong evidence to support the hypothesis that teacher self-efficacy has a positive effect on teacher well-being (Zee and Koomen, 2016; Huang et al., 2019; Ortan et al., 2021; Liang et al., 2022). According to Bandura’s (1986) social cognitive theory, self-efficacy is a key determinant of human behavior, as it influences the goals individuals set for themselves, the effort they put into achieving those goals, and their ability to persist in the face of obstacles and setbacks. When it comes to teaching, teachers with high levels of self-efficacy are more likely to set challenging goals for themselves, put in the necessary effort to achieve those goals, and persist in the face of obstacles, which in turn can lead to increased feelings of accomplishment and fulfillment (Tschannen-Moran and Hoy, 2001, 2007; Fathi et al., 2021).

*H3*: Teacher self-efficacy positively affects teacher enthusiasm.

Teacher self-efficacy is concerned with a teacher’s belief in their ability to perform their teaching role effectively (Tschannen-Moran and Hoy, 2007). Teachers with high levels of self-efficacy are more likely to take on new challenges, persist in the face of difficulties, and remain motivated in their work. These positive effects of teacher self-efficacy may also influence teacher enthusiasm (Lazarides et al., 2018, 2021; Michos et al., 2022). Enthusiasm is characterized as a strong feeling of interest or excitement toward a particular activity or goal (Keller et al., 2014). Teachers with high levels of self-efficacy may be more likely to feel enthusiastic about their work, as they have the confidence and belief in their ability to achieve success in their teaching role (Caprara et al., 2012; Lazarides et al., 2018, 2021; Michos et al., 2022). For instance, teachers with high levels of self-efficacy may be more likely to set challenging goals for themselves and feel confident in their ability to achieve those goals. This sense of accomplishment and satisfaction may then lead to increased enthusiasm and motivation in their work (Chan, 2011). In addition, teachers with high levels of self-efficacy may be more likely to seek out and engage in professional development opportunities (Postholm, 2012), which may lead to the acquisition of new skills and knowledge that can enhance their enthusiasm for teaching. Therefore, it seems warranted to propose that teacher self-efficacy has a positive effect on teacher enthusiasm.

*H4*: Teacher self-efficacy influences teacher grit positively.

As grit is described as perseverance and passion for long-term goals, teachers with high levels of grit are likely to persist in the face of challenges and setbacks, and to maintain their motivation and engagement over time (Fabelico and Afalla, 2020). Social cognitive theory (SCT) posulates that self-efficacy, or the belief in one’s ability to perform a task, is a critical determinant of behavior (Bandura, 1986). Teachers who have high self-efficacy beliefs are more likely to persist in the face of challenges, which is a key component of grit (Duckworth et al., 2009). In addition, teachers with high self-efficacy beliefs may be more likely to set challenging goals for themselves, and work tirelessly to achieve them, which are also important components of grit (Tschannen-Moran and Hoy, 2007).

Previous research has consistently shown that teacher self-efficacy is positively associated with a variety of positive outcomes, including motivation, job satisfaction, and well-being (Bandura, 1997; Tschannen-Moran and Hoy, 2007; Moè et al., 2010; Burić and Moe, 2020). Additionally, self-efficacy has been found to be a predictor of persistence and achievement in the face of obstacles (Lent et al., 1984; Bandura, 1997; Komarraju and Nadler, 2013; Garza et al., 2014), which is theoretically in accordance with the construct of grit. Also, recent investigations on the relationship between grit and self-efficacy have provided further support for this hypothesis. Sulla et al. (2022) found that grit was positively related to academic performance among university students during the COVID-19 pandemic, and Yang et al. (2022) demonstrated the contribution of academic buoyancy and self-efficacy to L2 grit among English language learners in Iran and China. These studies might suggest that grit and self-efficacy are positively related constructs and highlight the significance of investigating their joint influence on teacher well-being among EFL teachers. Therefore, it is likely that teacher self-efficacy positively influences grit, as teachers who believe in their ability to succeed are more likely to persist in the face of obstacles and to maintain their commitment to their goals (Dobbins, 2016; Sudina et al., 2021b; Zheng et al., 2022).

*H5*: Teacher enthusiasm has a positive influence on teacher grit.

Grit is defined as the combination of passion and perseverance toward long-term goals, and it has been shown to be a key predictor of success in various domains, including education (Duckworth and Seligman, 2005; Duckworth et al., 2007). Passion, as one component of grit, is characterized as an intense interest or enthusiasm toward a particular activity or goal, while perseverance is the ability to persist in that activity or goal despite setbacks or obstacles (Teimouri et al., 2022). Both passion and perseverance are crucial elements of grit and work together to drive individuals toward achieving their goals (Von Culin et al., 2014). In teaching contexts, enthusiasm can be seen as a manifestation of passion, and it can contribute to a teacher’s overall level of grit by enhancing their motivation and commitment to their goals (Kunter et al., 2011). Enthusiastic teachers are more likely to be passionate about their work, which can lead to higher levels of perseverance and persistence (Keller et al., 2016; Yang, 2021). They are also more likely to seek out and take advantage of opportunities to improve their teaching skills, which can further enhance their level of grit (Robertson-Kraft and Duckworth, 2014). From this perspective, it seems warranted to hypothesize that Teacher enthusiasm affects teacher grit positively.

*H6*: Teacher grit affects teacher well-being in a positive way.

It has been suggested that individuals with higher levels of grit are more likely to persist in the face of challenges, maintain their motivation and engagement over time, and ultimately achieve their goals (Duckworth et al., 2007). Teachers who possess higher levels of grit are more likely to handle the demands of the teaching profession, such as dealing with challenging students, navigating school policies, and balancing work and personal life, which can have a positive impact on their overall well-being (Bashant, 2014; Sudina et al., 2021a,b; Azari Noughabi et al., 2022).

Research evidence has also demonstrated that grit is positively associated with job satisfaction and engagement (Bashant, 2014; Dugan et al., 2019; Liu et al., 2023) and burnout (Halliday et al., 2017). From this perspective, teachers who have higher levels of grit are more likely to experience a sense of accomplishment and fulfillment in their work, which can lead to greater job satisfaction and higher levels of overall well-being. Additionally, teachers with high levels of grit might be better equipped to cope with the emotional demands of teaching (Ivcevic and Brackett, 2014), which can reduce the risk of burnout and improve overall well-being. As such, it is theoretically justifiable to propose that teacher grit positively affects teacher well-being by helping them handle the demands of the profession, experience a sense of fulfillment, and cope with the emotional demands of teaching.

## Methods

### Participants

To select the participants, the researcher employed convenience sampling, which involves selecting participants based on their accessibility, availability, and geographical proximity (Dörnyei and Csizér, 2012). The participants were 553 EFL teachers from senior high schools in China, representing ten provinces and two municipalities (Shanghai and Tianjin). The age of the participants ranged from 21 to 53 years, with 341 females and 212 males. The sample also included a diverse range of teaching experience: 131 (23.7%) had less than five years of experience, 93 (16.8%) had 5–10 years, 228 (41.3%) had 10–20 years, and 101 (18.2%) had over 20 years of experience. On average, the participants reported teaching 20 h per week, with a range of 12–28 h per week. The average class size reported by participants was 40 students, with a range of 30–50 students per class. The data were collected over a period of two months from March to April 2022.

Prior to participation, participants were informed about the study’s objectives and provided consent. Confidentiality and anonymity were ensured throughout the study, and participants had the right to withdraw from participation at any stage without providing a reason.

### Instruments

#### Teaching enthusiasm

The questionnaire developed by Kunter et al. (2011) was used to assess teacher enthusiasm. This questionnaire originally had 10 items that measured mathematics instructors’ teaching enthusiasm, but in this study, the items were modified by replacing “mathematics” with “English.” The participants rated each item on a 5-point scale, ranging from 1 (strongly disagree) to 5 (strongly agree). For example, an item from the scale is “I teach English with great enthusiasm.” In addition, previous studies have reported the reliability and validity of the scale. Kunter et al. (2011) reported a relatively high reliability (coefficient of Cronbach’s a = 0.83), while Kasalak and Dağyar (2021) also found evidence for the psychometric properties of the scale.

#### Teacher self-efficacy

The level of self-efficacy of Chinese EFL teachers was assessed using the Teachers’ Sense of Efficacy Scale (TSES) (Tschannen-Moran and Hoy, 2001). The TSES consists of 24 items, which are divided into three components: “Efficacy for Instructional Strategies,” “Efficacy for Classroom Management,” and “Efficacy for Student Engagement.” According to Tschannen-Moran and Hoy (2001), the reliability coefficients for the TSES subscales were high, with a score of 0.91 for instruction, 0.90 for management, and 0.87 for engagement. They also found that these three components also accounted for 54% of the variance in the teachers’ responses. TSES has been widely used in previous studies (e.g., Tsui and Kennedy, 2009; Duffin et al., 2012). Additionally, Klassen et al. (2009) verified the validity of the TSES scale in five countries. This scales uses a 5-point Likert scale, and participants respond to items on a scale ranging from 1 (nothing) to 5 (a great deal). The TSES is a reliable and validated scale that has been used in previous studies (e.g., Tsui and Kennedy, 2009; Duffin et al., 2012). A sample item from the TSES is “How well can you implement alternative strategies in your classroom?”

#### Psychological well-being at work

In this research, the Psychological Well-Being at Work (PWBW, Dagenais-Desmarais and Savoie, 2012), was employed to measure teachers’ psychological well-being. This questionnaire consists of five underlying facets which were reported to have high reliability coefficients by Dagenais-Desmarais and Savoie (2012): “Interpersonal Fit at Work (*α* = 0.920),” “Thriving at Work (*α* = 0.907),” “Feeling of Competency at Work (*α* = 0.861),” “Perceived Recognition at Work (*α* = 0.833),” and “Desire for Involvement at Work (*α* = 0.888).” The scale has 25 statements rated on a 6-point scale, ranging from 0 = *Disagree* to 5 = *Completely Agree*. This scale was chosen as a measure of teacher psychological well-being due to its comprehensive nature and ability to capture multiple dimensions of well-being in the workplace (Medzo-M’Engone and Ntsame Sima, 2021).

#### Grit

In addition, the study employed the Teacher L2 Grit Scale designed by Sudina et al. (2021b) to measure teachers’ L2 grit. The scale is a 14-item questionnaire rated on a 5-point Likert scale and measures two factors: consistency of interest (CI) and perseverance of effort (PE). The scale had acceptable reliability and validity indices as reported by Sudina et al. (2021b). Sudina et al. (2021b) reported acceptable internal consistency of ω = 0.77 for the scale, with ω = 0.69 for PE and 0.71 for CI. The Teacher L2 Grit Scale is a reliable and valid tool for measuring L2 grit among teachers (Liu et al., 2023). The items of the scale are measured on a 5-point scale varying from 1 (*not like me at all*) to 5 (*very much like me*). One sample item from the Teacher L2 Grit Scale is “I am a hardworking ESL/EFL teacher.”

### Data collection

Data for this study were collected using electronic questionnaires that were distributed through an online questionnaire system called “Questionnaire Star” in March and April of 2022. The questionnaires were initially sent to EFL teachers from senior high schools in various regions of China, who voluntarily agreed to participate in the study. Teachers were then requested to share the questionnaire with their colleagues *via* email and other social media platforms, such as Weibo and WeChat, to ensure a diverse sample. The questionnaire was specifically designed to collect data on teacher enthusiasm, teacher self-efficacy, grit, and psychological well-being, and took approximately 30 min to complete. Participants were informed about the research objectives and the confidentiality of their responses was guaranteed. Before participating, EFL teachers were notified of the study’s purpose and their right to withdraw from participation at any time without providing a reason. To increase response rates and reduce non-response bias, multiple reminders were sent to participants *via* email and social media platforms during the data collection period. Additionally, a small incentive (an electronic gift card) was offered to participants who completed the survey.

### Data analysis

As the first step, the researcher conducted descriptive and correlation analyses to explore the relationships among the factors using SPSS 23.0. To test the research hypothesis, Structural Equation Modeling (SEM) in Amos program (version 22.0) was employed. The measurement model was fitted to the data first (see Anderson and Gerbing, 1988), followed by an examination of the underlying structural model. To evaluate the overall fitness of the hypothesized model, several fit indices were employed. The fit indices included the *χ^2^*-goodness of fit to the degree of freedom (df) ratio, the Goodness of Fit Index (GFI), the Comparative Fit Index (CFI), the Root-Mean-Square Error of Approximation (RMSEA), and the Standardized Root-Mean-Square Residual (SRMR). The *χ^2^*/df was considered good if it was less than 3, with a value of *p* of greater than 0.05 (Chau, 1997). Additionally, fit indices such as GFI and CFI values of 0.90 or higher (Chau, 1997) indicate good fit, while RMSEA <0.08, and SRMR <0.10 (Vandenberg and Lance, 2000).

## Results

Table 1 presents the outcomes of the descriptive and correlation analyses, which revealed that EFL teachers displayed above-average levels of self-efficacy, enthusiasm, grit, and well-being. The correlation matrix indicated that all predictor variables were substantially associated with the dependent variable (i.e., well-being). Notably, teacher grit exhibited a strong and significant correlation with well-being (*r* = 0.527, *p* < 0.01). Likewise, teacher self-efficacy (*r* = 0.412, *p* < 0.01) and teacher enthusiasm (*r* = 0.289, *p* < 0.01) showed significant associations with well-being.

**Table 1:** Descriptive analysis.

	Mean	SD	1	2	3	4
1. Self-efficacy	3.12	0.815	1			
2. Enthusiasm	3.63	0.728	0.268**	1		
3. Grit	3.27	0.820	0.368**	0.384**	1	
4. Well-being	3.89	0.789	0.412**	0.289**	0.527**	1

**p-value < 0.01.

Then, an exploratory factor analysis was performed *via* maximum-likelihood technique with varimax rotation, incorporating all the indicators of the four latent constructs. The rotation matrix demonstrated that the cumulative percentage of variance accounted for by all four study variables was 48.26, providing evidence for the validation of the measurement scale.

Furthermore, a series of confirmatory factor analyses were conducted to test the unidimensionality of the latent factors, and three alternative measurement models were compared with the hypothesized baseline model. The results, as presented in Table 2, indicated that the hypothesized four-factor measurement model fit the data better than the alternative models, with *χ^2^* = 810.240, df = 523, *p* < 0.001, CFI = 0.974, GFI = 0.886, RMSEA = 0.029, and SRMR = 0.046.

**Table 2:** The results of measurement models.

Measurement Model	χ^2^	Df	CFI	GFI	RMSEA	SRMR
Single-factor model	985.324	527	0.949	0.859	0.043	0.257
Two-factor model	937.028	525	0.952	0.863	0.039	0.190
Three-factor model	886.419	524	0.961	0.871	0.034	0.113
Four-factor model	810.240	523	0.974	0.886	0.029	0.046

In addition to the confirmatory factor analysis results, the reliability of each construct was examined. Table 3 displays the Cronbach’s alpha coefficients and item-total correlations for each latent construct. The Cronbach’s alpha coefficients ranged from 0.851 to 0.911, indicating good internal consistency for each factor. The item-total correlations for all items exceeded 0.5, which indicates that the items measuring each construct are highly related to one another.

**Table 3:** Reliability of constructs and item-total correlations.

Construct	Cronbach’s alpha	Item-total correlation
Self-efficacy	0.851	0.79–0.88
Enthusiasm	0.862	0.67–0.81
Grit	0.911	0.75–0.86
Well-being	0.886	0.71–0.84

To establish convergent validity, the AVE was used as suggested by Fornell and Larcker (1981). Table 4 demonstrates that the AVE and CR of the constructs exceeded the threshold criteria of 0.50 and 0.6, respectively. All indicators of the baseline measurement model had loading higher than 0.5, which provided evidence of convergent validity. Discriminant validity was also evaluated using the criterion proposed by Straub et al. (2004), by comparing the square root of AVE with its related construct correlation. The results, presented in Table 5, revealed that the interconnection among all the factors was lower than the square root of AVE, confirming discriminant validity.

**Table 4:** Convergent validity and composite reliability.

	AVE	CR
1. Self-efficacy	0.57	0.925
2. Enthusiasm	0.56	0.901
3. Grit	0.51	0.898
4. Well-being	0.51	0.862

AVE, Average variance extracted; CR, Composite reliability.

**Table 5:** Discriminant validity.

	1	2	3	4
1. Self-efficacy	0.851			
2. Enthusiasm	0.771	0.826		
3. Grit	0.657	0.748	0.786	
4. Well-being	0.638	0.724	0.692	0.874

**Figure 1:**
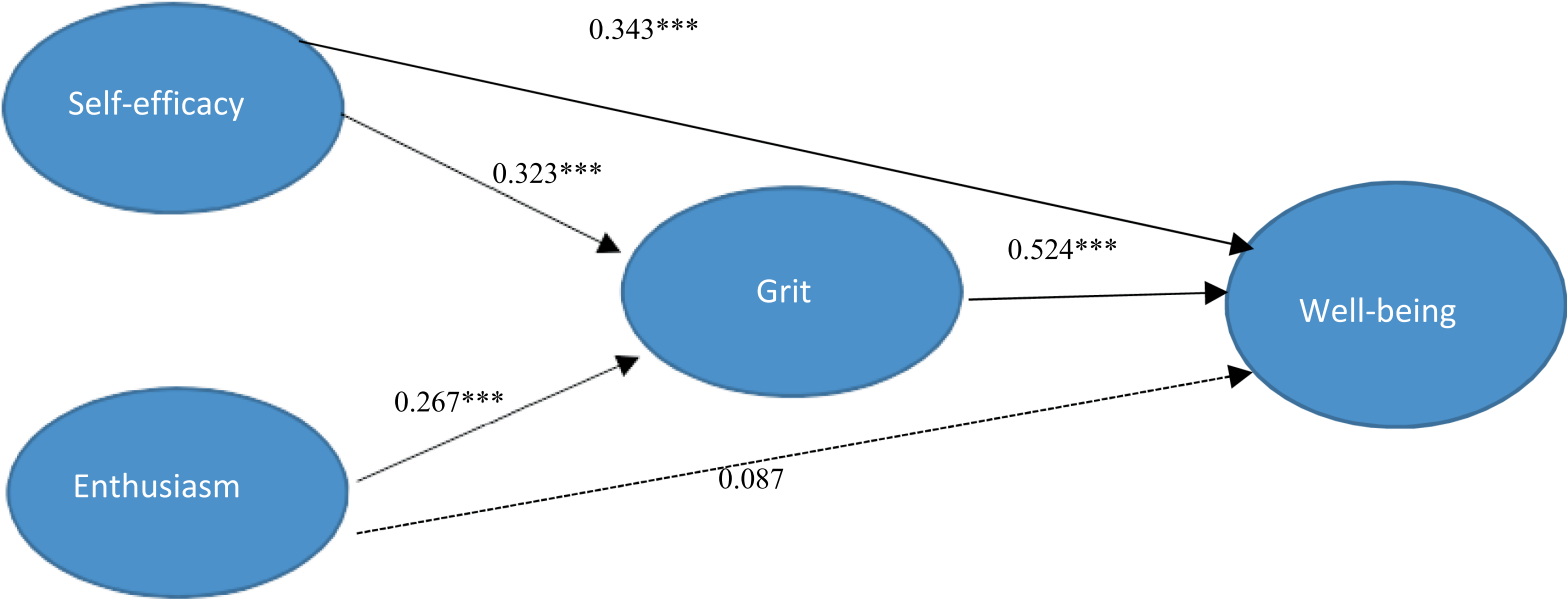
The Final Model.

After validating the measurement model, alternative structural models were examined to test the hypotheses. Specifically, the hypothesized partial mediation model (Model A) was compared with a full mediation model (Model B) and a direct model (Model C). The fit statistics of all three models are shown in Table 6. The hypothesized model (Model A) had a significantly better fit than Model B (Δdf = 5, Δχ2 = 87.22, *p* < 0.001) and model C (Δdf = 8, Δχ2 = 294.29, *p* < 0.001), based on the used fit indices. Thus, Model A was considered as the most parsimonious fit to the data. Figure 1 shows the path and parameter estimates for the final fit model (Model A). As seen in the Figure 1, all the path coefficients were significant except for the path between teacher enthusiasm and well-being. The structural model indicated that self-efficacy significantly influenced teacher grit (b = 0.323 *p* < 0.01). Similarly, teacher enthusiasm had a significant positive effect on teacher grit (b = 0.267, *p* < 0.01). Additionally, teacher grit was positively associated with well-being (b = 0.524, *p* < 0.01).

**Table 6:** Results of fit indices of structural models.

Model	χ^2^	df	Δχ^2^	GFI	CFI	RMSEA	TLI	SRMR
Direct Effect Model (C)	992.324 **	535	–	0.842	0.928	0.053	0.923	0.162
Full Mediation Model (B)	785.256 **	532	207.06	0.863	0.971	0.039	0.953	0.059
Partial Mediation Model (A)	698.034 **	527	87.22	0.881	0.981	0.029	0.979	0.051

Δ*χ*^2^ shows differences between model and the subsequent model. **p-value < 0.001.

Afterwards, Baron and Kenny's (1986) method was used to test whether teacher grit mediated the relationship among variables. The direct model (Table 7) revealed significant path coefficients between teacher self-efficacy, teacher enthusiasm, and well-being (self-efficacy → well-being: 0.373, *p* < 0.001; enthusiasm → well-being: 0.148, *p* < 0.05), which confirms the first step of Baron and Kenny’s method. The full mediation model found significant path coefficients between self-efficacy and enthusiasm on grit (self-efficacy → grit: 0.335, *p* < 0.001; enthusiasm → grit: 0.275, *p* < 0.01), which confirmed the second step of the method. The partial mediation model showed that teacher grit partially mediated the relationship between teacher enthusiasm and teacher well-being. In addition, teacher enthusiasm had an insignificant path coefficient on well-being, while teacher grit was a full mediator between teacher self-efficacy and teacher well-being. Thus, the influence of enthusiasm on teacher grit affected well-being. Finally, to account for common method bias, Harman’s single factor test was performed, including all latent variables assessed *via* self-reported measures (i.e., self-efficacy, enthusiasm, well-being, and grit). The first factor accounted for 23.41 percentage of variance that was lower than the threshold of 50%, indicating the absence of common method bias in this research.

**Table 7:** Path estimates of structural model.

Standardized path coefficients (*t*-value)			
	Direct effects model	Full mediation model	Partial mediation model
Self-efficacy → well-being	0.373 (5.12***)		0.343 (4.36***)
Enthusiasm → well-being	0.148 (2.32*)		0.087 (0.59)
Self-efficacy → grit		0.335 (4.36***)	0.323 (4.12***)
Enthusiasm → grit		0.275 (3.32**)	0.267 (3.24**)
Grit → well-being		0.682 (8.03***)	0.524 (7.37***)

*p-value < 0.05, **p-value < 0.01, ***p-value < 0.001.

## Discussion

The present study investigated the relationships among teacher enthusiasm, teacher self-efficacy, grit, and teacher well-being in the Chinese EFL context. The findings of the study revealed several important relationships that have implications for the promotion of teacher well-being in this context. Specifically, the study found that teacher self-efficacy had a positive direct effect on teacher well-being, that teacher grit had a positive direct effect on teacher well-being, and that teacher enthusiasm had an indirect effect on teacher well-being through the mediation of teacher grit.

The finding that teacher self-efficacy has a positive and direct effect on teacher well-being is consistent with previous research (Helms-Lorenz and Maulana, 2016; Zee and Koomen, 2016; Huang et al., 2019; Billett et al., 2022). Research indicates that teachers with stronger self-efficacy have better well-being, higher engagement, greater commitment to teaching, and lower levels of stress and burnout (Simbula et al., 2011; Malinen and Savolainen, 2016; Zee and Koomen, 2016; Li et al., 2017; Bing et al., 2022; Xiao et al., 2022; Xiyun et al., 2022). This is in line with Bandura’s (2006) assertion that self-efficacy is a primary factor influencing well-being and impacting personal and professional paths. Similarly, Federici and Skaalvik (2012) found that self-efficacy was negatively related to fatigue and positively related to motivation and job satisfaction. Instructors with low self-efficacy face more classroom challenges, higher levels of job stress, and lower job satisfaction (Betoret, 2006), which might contribute to their teaching effectiveness and well-being (Goddard et al., 2004). When teachers believe in their own abilities to manage their classrooms effectively, develop engaging and effective lesson plans, and engage in successful interactions with students and colleagues, they are more likely to feel competent and confident in their work (Liang et al., 2022). This sense of competence and confidence can contribute to a greater sense of satisfaction and fulfillment in their work, which can in turn lead to greater overall psychological well-being (Huang et al., 2019). Additionally, when teachers feel a sense of control over their work and believe that they have the skills and resources necessary to address challenges that arise, they are less likely to experience stress and burnout (Leijen et al., 2022). This is because they are better equipped to manage stressors and navigate difficult situations, which can ultimately lead to better psychological health.

Also, it was found that teacher well-being is positively impacted by teacher grit. This result is consistent with previous research that has demonstrated the importance of grit as a predictor of well-being in various settings, including education (Bashant, 2014; Sudina et al., 2021a; Azari Noughabi et al., 2022). Grittier teachers are more likely to persist when faced with challenges and find purpose and meaning in their work. It has been suggested that promoting EFL teachers’ well-being and enjoyment of teaching should be a priority in teacher education programs to prevent exhaustion, as teachers’ well-being is fundamental to their enjoyment of teaching (Mercer, 2020). Derakhshan et al. (2022) found that EFL teachers require mental well-being, resilience, and L2 grit to be happy, suggesting that resilient, gritty, and emotionally balanced teachers contribute to a positive classroom environment. Additionally, gritty teachers exhibit a strong commitment to their jobs and passion for their work, which can enhance their well-being (Maiers and Sandvold, 2017). Teachers with grit are also likely to have a growth mindset, which is positively associated with mental well-being and helps them cope with stressors (Zeng et al., 2019; Derakhshan et al., 2022). Overall, the study’s findings suggest that teachers with grit have a strong determination to achieve professional objectives and are less likely to experience exhaustion, resulting in higher levels of well-being.

Another finding of SEM analysis was that teacher enthusiasm has an indirect impact on teacher well-being through the mediation of teacher grit is a significant contribution to the field of teacher well-being. The study suggests that teacher enthusiasm has a positive impact on teacher grit, which, in turn, enhances the well-being of EFL teachers (enthusiasm → grit → well-being). This finding might be explained in light of some factors. First, enthusiastic teachers are more likely to be motivated and engaged in their teaching, which can lead to a greater sense of purpose and meaning in their work (Peng, 2021). This sense of purpose can help teachers to overcome obstacles and persist in the face of challenges (Leijen et al., 2020), which is a key component of grit (Robertson-Kraft and Duckworth, 2014). Second, enthusiastic teachers may be more likely to use a wider range of instructional strategies and be more responsive to students’ needs and expectations. This responsiveness can lead to more positive interactions with students, which can be rewarding for teachers and contribute to their overall sense of well-being (Manasia et al., 2020). Additionally, this finding that teacher enthusiasm has an indirect effect on teacher well-being through the mediation of teacher grit suggests that grit may play a key role in the relationship between enthusiasm and well-being. Specifically, teachers who are able to persist in the face of challenges and setbacks may experience a greater sense of accomplishment and satisfaction in their work, which can contribute to their overall well-being (Azari Noughabi et al., 2022).

Moreover, the interconnection between self-efficacy and grit has been found to be significant. Teacher self-efficacy, which is concerned with teachers’ beliefs about their own capability to accomplish specific tasks, plays a vital role in determining the activities teachers engage in, the amount of effort they put into such activities, and their perseverance in challenging situations. Several studies have found that teachers with higher levels of self-efficacy are grittier and employ a wide range of instructional strategies, and are more responsive to students’ needs and expectations than teachers who do not hold such beliefs (Mattern and Bauer, 2014; Kazemkhah Hasankiadeh and Azari Noughabi, 2022). Previous research has also shown that self-efficacy and self-regulation are two positive elements that can promote instructors’ self-determination and grit in the face of daily obstacles (Sudina et al., 2021a,b). Instructors with stronger self-efficacy are more likely to be dedicated and motivated throughout their careers (Skaalvik and Skaalvik, 2014). According to Sudina et al. (2021a), there may be theoretical correlations between language instructors’ grit and self-efficacy. They suggested that self-efficacy helps instructors use specific techniques for handling job-related obstacles and achieving professional goals. Strong self-efficacy can also boost instructors’ perseverance in the face of failure (Duckworth et al., 2009). This finding also aligns with Dobbins (2016) who investigated found that self-efficacy facilitates grit.

Finally, a positive association was found between teacher self-efficacy and teacher enthusiasm. Teacher enthusiasm is regarded as a way for teachers to demonstrate their passion for teaching and willingness to support student learning (Moe, 2016). Scholars often link motivation to self-efficacy, stating that motivation is a necessary component of self-efficacy for learning and performance (Hsieh and Schallert, 2008), as self-efficacy has the ability to influence one’s ideas and actions. The study also indicated that teacher efficacy is strongly related to other outcomes, including enthusiasm, which may be due to the fact that enthusiastic instructors tend to be happier, healthier, and more successful in their teaching (Keller et al., 2016; Taxer and Frenzel, 2018). Instructors who feel greater positive emotions at work had better degrees of enthusiasm, self-efficacy, and work satisfaction. Similarly, teachers who believe they can effectively manage difficult situations and feel content with their teaching work are more likely to experience enthusiasm (Burić and Moe, 2020). According to the broaden-and-build hypothesis (Fredrickson, 2001), favorable affective experiences extend individual thought-action repertoires and build persistent personal resources that support adaptive functioning and later emotional experiences. Thus, high emotional state felt at work helps instructors develop a feeling of efficacy and positive job-related perceptions, which are associated with a higher likelihood of experiencing and demonstrating enthusiasm in teaching.

Overall, the outcomes of this research have important implications for the promotion of teacher well-being in the context of EFL teaching. The study highlights the importance of promoting teacher self-efficacy, grit, and enthusiasm in order to support teacher well-being. In addition, the study provides evidence that teacher enthusiasm can have indirect effects on teacher well-being through the mediation of teacher grit, which suggests that interventions aimed at promoting teacher enthusiasm may have important benefits for teacher well-being in this context. These findings have important practical implications for the development of interventions and programs aimed at supporting teacher well-being in EFL teaching.

### Implications

The findings of this study have several theoretical and practical implications. Theoretically, the study provides evidence for the importance of teacher self-efficacy, grit, and enthusiasm in promoting teacher well-being in the context of EFL teaching. The study also contributes to the broader literature on teacher well-being, by highlighting the importance of specific teacher characteristics that can be targeted in interventions and programs aimed at promoting teacher well-being. Practically, the findings of this study have several implications for the development of interventions and programs aimed at promoting teacher well-being in the context of EFL teaching. The study highlights the importance of developing interventions that specifically target teacher self-efficacy, grit, and enthusiasm, in order to support teacher well-being. Interventions that focus on developing these characteristics may include training and development programs, mentoring and coaching programs, and mindfulness and stress reduction programs. In addition, the study suggests that interventions aimed at promoting teacher enthusiasm may have important indirect effects on teacher well-being through the mediation of teacher grit, which highlights the importance of interventions that specifically target teacher motivation and engagement.

The outcomes of this research have several theoretical and practical implications. Theoretically, the study provides evidence for the importance of teacher self-efficacy, grit, and enthusiasm in promoting teacher well-being in the context of EFL teaching. The study also contributes to the broader literature on teacher well-being, by highlighting the importance of specific teacher characteristics that can be targeted in interventions and programs aimed at promoting teacher well-being. It is worth noting that there is a growing body of research that demonstrates the effectiveness of various interventions and programs aimed at promoting teacher self-efficacy, grit, and enthusiasm. For example, coaching programs have been found to be effective in increasing teacher self-efficacy (Johnson and Birkeland, 2003; Goker, 2006), while mindfulness-based stress reduction programs have been shown to increase teacher well-being and decrease stress (Roeser et al., 2013). Additionally, mentoring and coaching programs have been found to be effective in promoting teacher grit (Blase and Kirby, 2008; Robertson-Kraft and Duckworth, 2014). As such, it is suggested that interventions and programs aimed at promoting teacher well-being should target teacher self-efficacy, grit, and enthusiasm. These interventions may include training and development programs, mentoring and coaching programs, and mindfulness and stress reduction programs. Furthermore, to foster enthusiasm and grit in teachers, it is important to provide them with opportunities to engage in activities that promote their sense of purpose, autonomy, and mastery (Deci and Ryan, 2008). For instance, providing instructors with opportunities for professional development and collaboration, and recognizing and rewarding their achievements can be effective strategies for promoting teacher motivation and engagement, and ultimately, teacher well-being.

This research might have some limitations that should be taken into account while interpreting the results. First, the study used a cross-sectional design, which limits the ability to draw causal inferences. Longitudinal designs would be necessary to make causality inferences between the variables under investigation. Future research could use longitudinal designs to establish causal relationships between teacher self-efficacy, grit, enthusiasm, and well-being in the context of EFL teaching. Second, the study used self-report measures, which are subject to bias and may not accurately reflect the true state of the variables under investigation. Future research could use objective measures of teacher well-being, such as physiological markers or observational measures, to supplement self-report measures and provide a more comprehensive understanding of the variables under investigation.

Also, future researchers could use experimental designs to test the effectiveness of interventions aimed at promoting teacher self-efficacy, grit, and enthusiasm, and to examine the mechanisms through which these interventions may affect teacher well-being. Third, the study was conducted in a specific cultural and linguistic context (EFL teaching), which limits the generalizability of the findings to other contexts. Future research could examine whether the relationships among teacher self-efficacy, grit, enthusiasm, and well-being differ across different cultural and linguistic contexts. Finally, further empirical studies might examine the role of other variables that may affect teacher well-being in the context of EFL teaching, such as job demands, job resources, and social support.

## Data availability statement

The data analyzed in this study is subject to the following licenses/restrictions: The raw data supporting the conclusions of this article will be made available by the author, without undue reservation. Requests to access these datasets should be directed to GS, shaoguohua888@163.com.

## Ethics statement

The studies involving human participants were reviewed and approved by Institute of Physical Education, Inner Mongolia Normal University, Hohhot. The patients/participants provided their written informed consent to participate in this study.

## Author contributions

The author confirms being the sole contributor of this work and has approved it for publication.

## Conflict of interest

The author declares that the research was conducted in the absence of any commercial or financial relationships that could be construed as a potential conflict of interest.

## Publisher’s note

All claims expressed in this article are solely those of the authors and do not necessarily represent those of their affiliated organizations, or those of the publisher, the editors and the reviewers. Any product that may be evaluated in this article, or claim that may be made by its manufacturer, is not guaranteed or endorsed by the publisher.
